# Safety data of Montanide ISA 51 VG and Montanide ISA 720 VG, two adjuvants dedicated to human therapeutic vaccines

**DOI:** 10.1186/2051-1426-3-S2-P428

**Published:** 2015-11-04

**Authors:** Stephane Ascarateil, Aude Puget, Marie-Eve Koziol

**Affiliations:** 1 SEPPIC, Puteaux, France; 2 SEPPIC Inc, Fairfield, NJ, USA

## Introduction

Montanide ISA 51 VG and Montanide ISA 720 VG are two novel adjuvants dedicated to human therapeutic vaccines. They have been used in more than 200 clinical trials involving cancer, AIDS, malaria or autoimmune disease vaccines and involving an accrual of more than 20000 patients. The adjuvants are rendering stable water-in-oil emulsions when mixed with water based antigenic media. Montanide ISA 51 VG is based on a blend of mannide monooleate surfactant and mineral oil, whereas Montanide ISA 720 VG uses a non-mineral oil. Although they have shown preclinical safety, a survey on clinical environment is performed.

## Material and methods

112 published clinical trials with both adjuvants are reviewed and number of patients per indications are noticed, as well as the parameters of vaccination such as route of innoculaion, volume, antigen dose, or co-stimulating agents. Safety data obtained are reviewed and are classified according to their nature, type, and intensity.

## Results

Montanide ISA 51 VG was mainly used in cancer and aids vaccination trials, with an accrual of 6000 patients. In cancer field the main represented projects are on melanoma and lung cancer, from clinical Phase I to Phase IV. Doses can be repeated as up to 10 per year and per patient, and are mainly given subcutaneously. Local and general adverse events observed with the vaccines are mild to moderate and generally transient, and refer to headache, local pain or redness at injection site. Placebo injections do not lead to adverse reactions. Montanide ISA 720 VG was used in 2000 people, mainly healthy volunteers of malaria vaccines projects. Injection is mainly intramuscular, and redness, granulomas or pain are described. This adjuvant was also used in cancer projects more recently.

## Conclusion

Montanide ISA 51 VG and Montanide ISA 720 VG are novel adjuvants intended to be used in therapeutic vaccination programs known to be potent adjuvants inducing CD4 and CD8 responses. This safety review demonstrates that they are safe and are suitable for registration by agencies like for NSCLC vaccine in 3 countries

